# An application of machine learning with feature selection to improve diagnosis and classification of neurodegenerative disorders

**DOI:** 10.1186/s12859-019-3027-7

**Published:** 2019-10-11

**Authors:** Josefa Díaz Álvarez, Jordi A. Matias-Guiu, María Nieves Cabrera-Martín, José L. Risco-Martín, José L. Ayala

**Affiliations:** 1 Dep. of Computer Architecture and Communications, Universidad de Extremadura, Mérida-Badajoz, Spain; 2 Dep. of Neurology, Hospital Clinico San Carlos, San Carlos Research Health Institute (IdISSC), Universidad Complutense, Madrid, Spain; 3 Dep. of Computer Architecture and Automation, Universidad Complutense, Madrid, Spain

**Keywords:** Machine learning primary progressive aphasia, Supervised algorithm, Unsupervised algorithm, Clustering Analysis

## Abstract

**Background:**

The analysis of health and medical data is crucial for improving the diagnosis precision, treatments and prevention. In this field, machine learning techniques play a key role. However, the amount of health data acquired from digital machines has high dimensionality and not all data acquired from digital machines are relevant for a particular disease. Primary Progressive Aphasia (PPA) is a neurodegenerative syndrome including several specific diseases, and it is a good model to implement machine learning analyses. In this work, we applied five feature selection algorithms to identify the set of relevant features from 18F-fluorodeoxyglucose positron emission tomography images of the main areas affected by PPA from patient records. On the other hand, we carried out classification and clustering algorithms before and after the feature selection process to contrast both results with those obtained in a previous work. We aimed to find the best classifier and the more relevant features from the WEKA tool to propose further a framework for automatic help on diagnosis. Dataset contains data from 150 FDG-PET imaging studies of 91 patients with a clinic prognosis of PPA, which were examined twice, and 28 controls. Our method comprises six different stages: (i) feature extraction, (ii) expertise knowledge supervision (iii) classification process, (iv) comparing classification results for feature selection, (v) clustering process after feature selection, and (vi) comparing clustering results with those obtained in a previous work.

**Results:**

Experimental tests confirmed clustering results from a previous work. Although classification results for some algorithms are not decisive for reducing features precisely, Principal Components Analisys (PCA) results exhibited similar or even better performances when compared to those obtained with all features.

**Conclusions:**

Although reducing the dimensionality does not means a general improvement, the set of features is almost halved and results are better or quite similar. Finally, it is interesting how these results expose a finer grain classification of patients according to the neuroanatomy of their disease.

## Background

Learning from data is one of the most successful fields applicable to many heterogeneous areas and disciplines like statistics, artificial intelligence, engineering, health, etc. Digital machines such as Magnetic Resonance Imaging (MRI), Mass Spectrometry (MS) or Positron Emission Tomography (PET), among others, and new generation sensors, have found their way in biomedical systems. In the recent decades, a plethora of advances in biotechnology have increased the diagnosis precision and the efficiency with tailored treatments. Furthermore, these machines generate wealth of data, which must be analyzed to extract the valuable knowledge that they contain. Data mining and machine learning techniques [1] are crucial to explore, extract and interpret the generated data. *Bioinformatics* as a multidisciplinary area includes the application of computational tools to biological, medical or health data acquired [2]. During the last decade, the amount of data has exponential growth, and the need to extract new knowledge for tackling diseases places bioinformatics as a priority research area. In this regard, machine learning and big data methods have been applied to better understand and fight many diseases [3–6].

The results of data analysis allow to identify patterns and develop predictive models to determine future behaviors in a particular context. In a biomedical scenario, machine learning techniques will provide an informed decision making, which will also have positive consequences for the health of patients. It is possible to implement a predictive model from a set of heterogeneous data acquired from diverse patients, which can be used to predict accurately the outcome for a new set of instances.

A major concern from a health and economic point of view is the impact of the aging population in the healthcare system, which is being studied and documented in all countries. Neurodegenerative diseases affect a large percentage of the old population and, to date, there is no cure for these diseases. Researchers focus on discovering new treatments for slowing down their progress and improve the quality of life of the patients. However, the success of clinical trials is hampered by a wide heterogeneity in clinical, genetic and pathological characteristics of neurodegenerative disorders [7].

Machine learning and data mining techniques can help to know the disease evolution and personalize treatments according to the needs and outcome of each patient. Personalized treatments rely on a proper diagnosis, where a specific subtype of the disease can be determined by subtle details in the imaging tools, and rule the prognosis. In this area, identifying the best classifier and clustering method will be the first step. However, taking a decision about the best classifier or clustering method to be used for reaching the most accurate results is a complex task. On the other hand, not all features are equally relevant in order to identify a disease or the stage of its evolution.

In this paper, we focus on Primary Progressive Aphasia (PPA). PPA is a clinical syndrome characterized by the neurodegeneration of language brain networks [8]. Generally, PPA is the clinical onset of specific and frequent neurodegenerative diseases such as Alzheimer’s disease, tauopathies, or TDP-43 proteinopathies, which represent the main pathological subtypes of neurodegenerative disorders. Intriguingly, three clinical variants have been described in PPA (ie. nonfluent, semantic, and logopenic PPA), and each variant impairs certain brain regions and is more or less suggestive of a pathological subtype [9]. In this regard, non-fluent or agrammatic variant is associated to left frontal lobe damage and is suggestive of tauopathies or less frequently TDP-43 proteinopathies; semantic variant is characterized by anterior temporal lobe impairment and is highly suggestive of TDP-43 type C pathology; and in logopenic variant, left parieto-temporal lobe is involved and it often precedes Alzheimer’s disease [10, 11]. This heterogeneity regarding the underlying pathology and different clinical courses [12] in patients with a similar clinical presentation (ie. aphasia) makes PPA a good model among neurodegenerative disorders to apply the machine learning techniques. Indeed, we recently have introduced the possibility of the existence of some additional subtypes within non-fluent and logopenic variants, with a better prediction of outcome using clustering analysis [13]

Diagnosis of neurodegenerative disorders and, in particular PPA, has been improved with the use of advanced neuroimaging techniques [14]. Among them, PET with 18F-fluorodeoxyglucose (FDG-PET) is a marker of synaptic dysfunction and reflects the brain topography of neurodegeneration with the measurement of brain metabolism [15]. As a consequence, it is considered a useful, early and reliable tool in the diagnostic assessment of cognitive disorders and PPA, because each neurodegenerative disease trends to impair specific brain regions [16]. However, the assessment of brain metabolism requires experience. Inter-rater agreement for visual analysis of PET imaging is usually moderate, but it could be improved with the use of some semiautomatic and statistical techniques [11, 17].

The purpose of our work is to propose a machine-learning based framework suitable for the analysis of FDG-PET images of PPA patients, in order to solve two critical questions: i) to help on the automatic diagnosis of this disease; ii) to identify subtypes of the illness that have correlations with the anatomy of the damaged brain.

In [18], the authors applied Hierarchical clustering according to the clinical language deficit to classify patients. As a result, two groups were identified corresponding to nonfluent aphasia and semantic dementia. [19] studied the association between the PPA severity and the disease duration, and disease duration and temporoparietal athophy on patients diagnosed of logopenic variant of primary progressive aphasia (lvPPA). They identified three variants of lvPPA using Hierarchical clustering on language, and neurocognitive test scores.

To perform this work, we used two data sets from 150 FDG-PET brain images, which comprised 116 brain attributes belonging to the normalized metabolism of brain regions and three more attributes, which are sex, age and clinical prognosis. Characteristics of participants and data acquisition methodology are explained as follows.

### Participants

This study involved 150 FDG-PET imaging studies belonging to 91 patients with PPA (31 of them were examined twice in two different moments of the disease) and 28 healthy controls.

PPA is a rare neurodegenerative syndrome and thus, 91 patients in PPA is considered a large sample, even more considering that all patients were studied with a comprehensive protocol including FDG-PET. Regarding this, in the recent joint effort of the European Association of Nuclear Medicine and the European Academy of Neurology [20] to address the utility of FDG-PET in the assessment of primary progressive aphasia, four articles with PPA and FDG-PET were selected. The sample size of these works was in all cases <55. Although PPA is a rare disease, we chose to study this disorder because it is a good model of brain-behaviour relationships, and it may be the onset of different clinical-pathological neurodegenerative entities.

PET imaging was performed in single-center between November 2011 and May 2017. All PPA patients met the current consensus criteria [21] and were classified into the three clinical variants taking into account the diagnostic criteria and follow-up. Further details about clinical and neuropsychological assessments are shown in a previous work [13].

The Institutional Research Ethics Committee from Hospital Clinico San Carlos approved the research protocol.

### FDG-PET imaging acquisition and preprocessing

PET images were acquired following European guidelines [22]. All images were obtained in the same scanner, which is a Siemens Biograph True Point PET-CT that integrates a 6-detector CT with a late-generation PET using lutetium oxyorthosilicate crystals. A mean dose of 185 MBq was administered 30 minutes before the acquisition of the images and after at least 6 hours of fasting. Patients remained at sensory rest prior to the acquisition. CT scan parameters were: kVp/effective mAs/rotation: 130/40/1; slice thickness: 3 mm; reconstruction interval: 1.5 mm; and pitch: 0.75. Acquisition time was 10 min.

Statistical Parametric Mapping software was used for imaging preprocessing (http://www.fil.ion.ucl.ac.uk). Images were realigned and normalized to the Montreal Neurological Institute standard space using a specifically developed brain FDG-PET template for dementia [23]. Global mean normalization was performed individually for intensity scaling. *Marsbar* software was used to perform a region of interest analysis, calculating the mean uptake value of each participant for the 116 brain areas of the Automatic Anatomical Labeling atlas belonging to the whole brain.

The rest of this paper is organized as follows. Experimental results are analyzed in “Results” section. Results discussion is presented in “Discussion” section and conclusions are drawn bellow. “Methods” section describes the methodology applied in this work.

## Results

The software toolkit WEKA was used in our experimental approach. WEKA is available in http://www.cs.waikato.ac.nz/ml/weka as a complete industrial-strength software for machine learning. Analyzed data come from FDG-PET images of each obtained cluster and were compared to an additional control group of 32 healthy subjects. Previously to be entered in the statistical analysis, images had been spatially normalized and smoothed at 12 mm full-width at half maximum. Statistical Parametric Mapping version 8 was used for preprocessing and analysis. A two-sample T test was conducted to compare between groups, using age and gender as covariates. Statistical significance was set at *p*<0.05 using family-wise error correction at cluster level.

For exploring the dataset, K-Fold Cross Validation was used as testing methodology. K-fold cross validation avoids overlapping by splitting data into *k* subsets and makes *K* iterations. For each iteration, a different subset was chosen for testing and the remainder for training. We picked *k*=10 because this value is considered appropriate to obtain an accurate estimation.

**Fig. 1:**
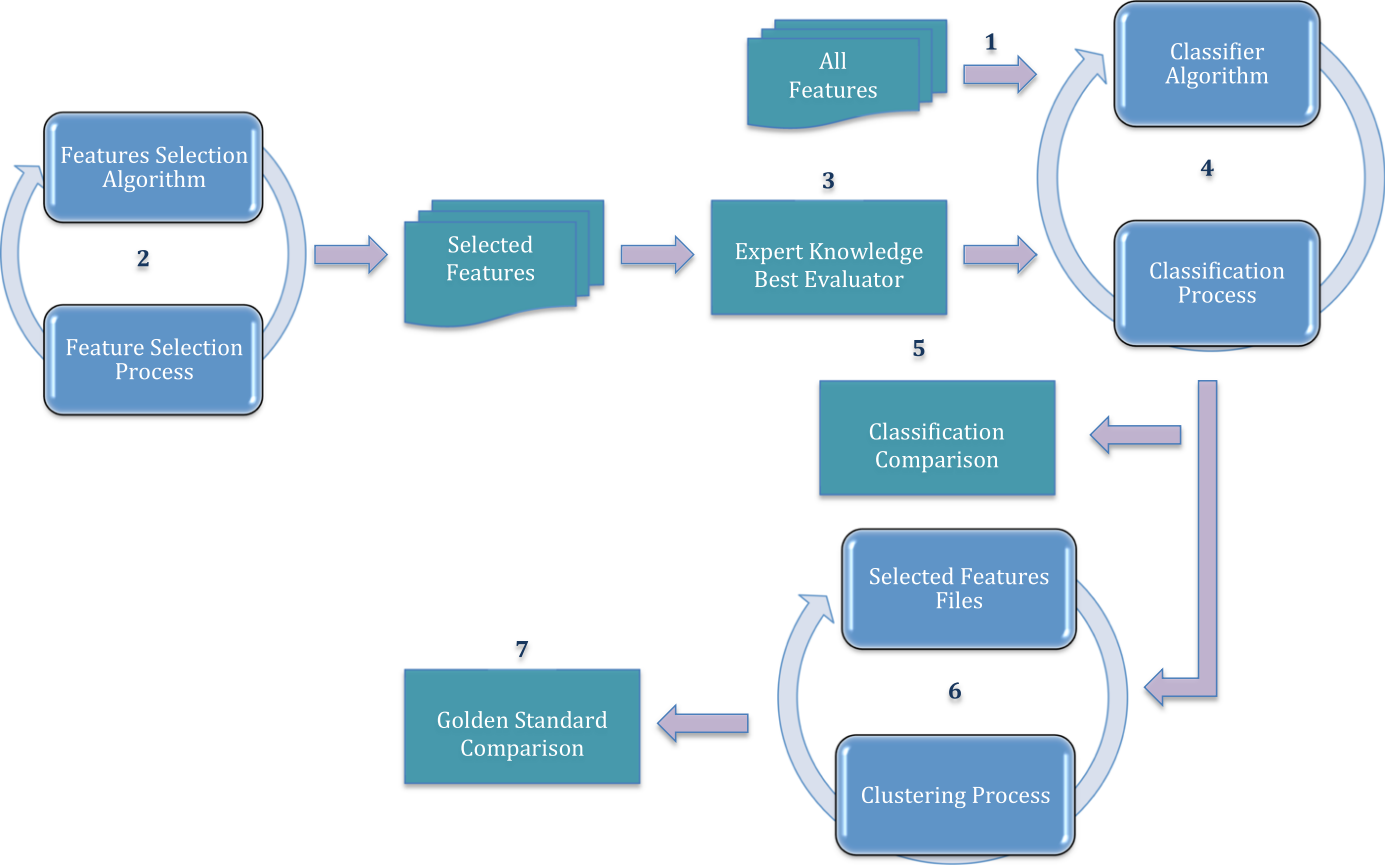
Methodology flow chart. Methodology applied divided into 6 different phases. Feature selection, classification, expertise knowledge, clustering and results comparison

**Fig. 2:**
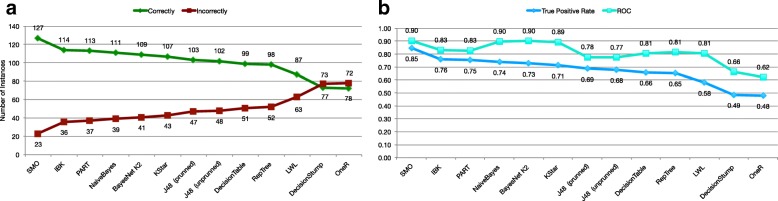
Classification with all features. **a** represents the number of instances correctly and incorrectly classified for each evaluated algorithm. **b** graphs the True Positive rate and the ROC metric for each evaluated algorithm

Our first experimental work, which corresponds to the phase 1 in the flow chart in Fig. 1, compared a set of supervised learning algorithms in order to find the best classifier. In this step, we considered the fully set of brain attributes. Figure 2a shows the classification results obtained for each evaluated classifier. Figure 2b shows the True Positive rate and the Receiver Operating Characteristics metric (ROC) for each algorithm. ROC is an independent measure to evaluate the performance of a prediction model, that relates the True Positive Rate (TPR) against to the False Positive Rate (FPR). The optimal prediction model will have a ROC value of 1. Data were sorted by the number of instances correctly and incorrectly classified and the True Positive rate (TP rate), respectively. According to these results, *SMO* algorithm presents the best performance with 127 instances correctly classified and 23 incorrectly classified (with respect to the clinical diagnosis criteria). *SMO* is followed by *IBK* algorithm with 114 instances correctly classified and 36 instances incorrectly classified.

Looking at *PART* and *Bayesian Naives* classifiers, *PART* obtained similar results to *IBK* with 113 and 37 instances correctly and incorrectly classified, respectively. *Naives* is the third best classifier, with 111 and 39 instances correctly and incorrectly classified, respectively.

Regarding the ROC metric, *SMO*, *BayesNet* and *Naives* presented the best performance with a ROC value of 0.9, followed closely by *Kstar* with 0.89, the closest values to 1, the optimal value. A good performance was obtained by *IBK* and *PART* with 0.83.

According to these results, we selected *SMO* as the best among those evaluated and *IBK* as the second one. However, as aforementioned, this approach used all the attributes for classification, while not all of them have the same weight neither provide relevant information for PPA diagnosis. Therefore, it is interesting to analyze which features are most important and provide more information for an accurate diagnosis. For that purpose, we developed a feature selection process by evaluating the algorithms presented in next section.

### Feature selection results

The feature selection process, identified as the phase 2 in the flow chart, was launched and for each feature selection algorithm a new dataset was obtained that comprises only the features considered as most relevant by every algorithm. In the particular case of PCA, we tested different number of PCs in order to achieve accurate results. *ChiSquaredAttributeEval* and *ClassifierAttributeEval* generate a ranked dataset, where each feature is ranked by their merit value. We picked those with a merit greater than 0 for generating the new subset. The two other algorithms return directly a dataset with the subset of most relevant features according to the specific implementation.

Algorithms *CfsSubsetEval* and *WrapperSubsetEval* apply *Best First* as the search method and return the number of subsets evaluated and an internal metric used by both algorithms to choose the best subset. 3314 and 1258 subsets were evaluated, until the best one was found, in the case of the *CfsSubsetEval* and *WrapperSubsetEval*, respectively.

At this step, we incorporate the expert knowledge and the subsets of features selected by the algorithms are presented to an experienced neurologist who is required to apply his criteria. This step is represented in the flow chart as the phase 3. The neurologist is able to evaluate the coherency of the features returned by each algorithm with respect to the neuroanatomy of the disease. Although this step can be easily automatized by a set of rules that categorize the brain regions that can be damaged by APP variants, we decided to present a clear a meaningful organization of selected features to the neurologist in order to acquire their knowledge without further noise.

In our case, the neurologist examined the results obtained for each algorithm. For the *CfsSubsetEval* algorithm, he considered the use of **right angular** as justifiable, however the algorithm immediately selected the **cerebellum cruss** and **posterior cingulum**, which is directly associated to logopenic variant. Moreover, **cuneus** is too posterior for three standard variants and next selected features get into the frontals. Hence he ruled out *CfsSubsetEval* as the most promising for the next phase.

With respect to *WrapperSubsetEval*, he considered that starting with the **left calcarine** is not a good option. This area is too posterior and it is not associated to language regions. Then, it follows in a right way, but it also uses the **occipital** area, which is also found very posterior and more linked to visual function. The expert dismissed the dataset resulting for this algorithm.

*ChiSquaredAttributeEval* and *ClassifierAttirbuteEval* were presented as two really reasonable proposals and the datasets could be used for the next phase. They have lots of similarities. However, *ChiSquaredAttributeEval* included many temporal areas before other regions were included. Moreover, the **occipital** regions were included before the **frontal** regions. Hence, it was finally dismissed.

The neurologist considered the features selected by the *ClassifierAttributeEval* algorithm, shown in Table 1, as the most suitable option. The main reason was the high ratio of attributes selected between the different **lobes**. Thus, the dataset resulting from this algorithm will be used in the following classification and clustering phases.

**Table 1:** Features for ClassifierAttributeEval algorithm sorted by relevance

ClassifierAttributeEval				
% Feature	% Feature	% Feature	% Feature	% Feature
0.144 TemporalPole Mid L	0.087 Parahippocamp L	0.055 Occipital Mid R	0.031 Supramarginal R	0.015 Paracentrallobule LM
0.137 SMA L	0.080 Occipital Mid L	0.051 Frontal Sup Medial L	0.031 Precuneus L	0.015 Precentral R
0.135 TemporalInf L	0.077 CingulumPost L	0.050 Cerebellum 8 L	0.030 Cerebellum 9 L	0.010 Rolandic Oper R
0.133 Fusiform L	0.077 TemporalSup L	0.049 Frontal Sup R	0.029 Insula R	0.010 Cerebellum 8 R
0.123 SMA R	0.075 Cerebellum 6 R	0.049 CingulumAnt R	0.027 ParietalInf L	0.010 Olfactory R
0.117 Occipital Inf L	0.072 Cuneus L	0.047 Frontal Inf Tri L	0.024 Insula L	0.010 ParietalSup L
0.110 CingulumAnt L	0.067 Frontal Sup L	0.041 TemporalMid R	0.023 Occipital Sup L	0.007 Paracentrallobule R
0.100 Angular R	0.064 Vermis 8	0.037 Supramarginal L	0.021 Frontal Mid R	0.003 Poscentral L
0.097 TemporalMid L	0.059 Frontal Inf Oper L	0.037 Hippocampus L	0.019 Thalamus L	0.001 Frontal Inf Orb L
0.093 Angular L	0.057 Precentral L	0.037 TemporalInf R	0.019 Heschl L	0.000 Sexo

Principal Components (PC) as a result of PCA is a different mater. Each PC is an uncorrelated variable consisting on a number of probably correlated variables and it covers a part of the data variance.Table 2 shows the PCs with an eigenvalue higher than 1, placed in the second column, the third column corresponds to the proportion in the total, the cumulative value is displayed in the fourth column, and finally the features included. Features chosen belong to different PCs in order to launch the classification process again and analyse its results. Repeated features trough different PCs were selected only once.

**Table 2:** Principal components of PCA with eigenvalue higher than 1

Principal components analyses								
PC	EigenValue	Proportion	Cumulative	F1	F2	F3	F4	F5
1	29.385	0.249	0.249	Frontal Mid Orb L	Frontal Inf Orb L	CingulumAnt L	Frontal Inf Tri L	Frontal Sup Orb L
2	19.402	0.164	0.413	Occipital Mid L	Frontal Inf Orb R	TemporalMid L	Fusiform L	Occipital Inf L
3	13.378	0.113	0.527	Precentral R	Frontal Mid R	ParietalInf R	Poscentral L	Poscentral R
4	7.585	0.064	0.591	TemporalPole Sup R	Parahippocamp R	Hippocampus R	TemporalPole Mid R	Precentral L
5	6.411	0.054	0.645	Thalamus R	Heschl L	Calcarine L	Heschl R	Cerebellum Crus1 L
6	4.807	0.041	0.686	TemporalInf R	TemporalMid R	EdadPET	Rectus R	Frontal Sup Orb R
7	3.946	0.033	0.720	Rolandic Oper L	Thalamus R	CingulumPost R	Poscentral L	TemporalPole Mid L
8	3.318	0.028	0.748	Pallidum L	Thalamus L	Vermis 10	Pallidum R	Putamen L
9	2.845	0.024	0.772	Sexo	Rolandic Oper L	Rolandic Oper R	Heschl R	Paracentrallobule R
10	2.314	0.020	0.791	CingulumPost R	CingulumPost L	Vermis 10	Caudate R	Cerebellum 9 R
11	2.279	0.019	0.811	Vermis 10	SMA L	Cerebellum Crus1 R	Rectus R	Pallidum L
12	1.779	0.015	0.826	Amygdala R	TemporalPole Mid R	Amygdala L	Sexo=2	Vermis 9
13	1.674	0.014	0.840	Vermis 1 2	ParietalSup L	Calcarine L	ParietalInf R	Cerebellum 3 L
14	1.460	0.012	0.852	Cerebellum 10 R	Cerebellum 10 L	Amygdala L	Cerebellum 3 R	Amygdala R
15	1.156	0.010	0.862	Olfactory R	Olfactory L	TemporalPole Sup R	TemporalPole Sup L	Sexo
16	1.122	0.010	0.872	Putamen R	Putamen L	Cerebellum Crus2 R	Parahippocamp R	Frontal Inf Oper R
17	1.066	0.009	0.881	Amygdala R	Cerebellum 10 R	Amygdala L	Cerebellum 10 L	TemporalInf R

These results are analysed by the expert neurologist, which presented as the most relevant the principal components ranging between 1 to 8, and 10 to 15. These are chosen for the next phase.

### Classification after feature selection

**Fig. 3:**
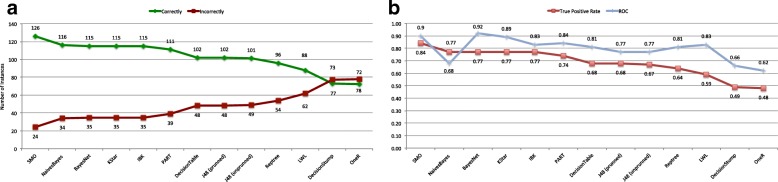
Classification after feature selection except PCA. **a** represents the number of instances correctly and incorrectly classified for each classification algorithm and the set of features selected above. **b** graphs the True Positive Rate and the ROC metric for each classification algorithm and the set of features selected above

Data subset obtained in the feature selection process and chosen by the neurologist has been provided as input to the classification process under the same conditions as the first experimental work. Figure 3a shows for the feature selection algorithm chosen in the previous phase, and classifiers addressed, the number of instances correctly and incorrectly classified. Figure 3b represents the evolution of the True Positive rate and the ROC metric for each feature selection algorithm and classifiers.

We analyzed these new results and compared them to those with all features. Regarding results after feature selection, the *SMO* algorithm is presented as the best classifier with 126 instances correctly classified, only one less than with all features. *Naives* ranks second behind *SMO* with 116 instances correctly classified, and *Bayesnet*, *Kstar* and *IBK* classified correctly 115 instances.

Comparing classification before and after feature selection, 6 out of 13 analyzed classifiers improved their results (*IBK*, *Naives*, *BayesNet*, *Kstar*, *DecisionTable*, *LWL*), 2 obtained identical results (*DecisionStump* and *OneR*), while results for 5 algorithms got worse (*SMO*, *Part*, *J48 (prunned)*, *J48 (unprunned)* and *RepTree*). From the performance perspective of the classification model using the ROC metric, *BayesNet* improved with a value of 0.92, the best one, followed closely by *SMO* and *Kstar* with 0.9 and 0.89, respectively. *PART* and *IBK* presented good performance with 0.84 and 0.83, improved and sustained their previous performance values. However, *Naives* obtained a poor value 0.68 compared to the previous one 0.9.

After this analysis, we cannot conclude that feature selection improves the number of instances correctly classified because the improvement percentage is not meaningful, although results are promising regarding the performance reached by some classifiers.

**Fig. 4:**
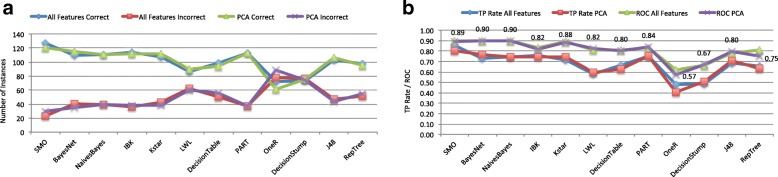
Classification after PCA analyses. Graphs detail the results obtained for PCs ranges 1-8 and 10-15. Figure 4**a** details the number of instances correctly and incorrectly classified vs. classification results with all features. Figure 4**b** compares both true positive rates and ROC metric

Regarding PCA results obtained in the classification process and shown in Fig. 4, it is difficult to generalize that PCA improves classification. However, experimental results demonstrated that some classification algorithms are able to improve or at least equal the number of instances correctly classified with a less number of features. Therefore, *BayesNet* obtains an improvement of 6 instances. *Naives* and *Part* equal previous results. *Kstar* classifies correctly 5 more than original tests, while *LWL*, *DecisionStump* and *J48* improves previous results in 3 instances. This is an interesting result as we can achieve similar classification performance from a reduced number of features, hence simplifying the selection criteria by a set of rules that relate brain anatomy and PPA.

Finally, *SMO*, *IBK*, *DecisionTable*, *OneR* and *RepTree* obtain worse results. Moreover, these results were reached with less than half of the features 55 out 119. However, *SMO* obtained the best result with 120 instances correctly classified. Moreover, *SMO*, *BayesNet*, *Naives* obtained ROC values close to the optimal 1, 0.89, 0.9, 0.9, respectively. *Kstar* obtained good performance with 0.88, *PART* improved with 0.84 and *IBK* got a little worse from 0.83 to 0.82.

Nevertheless and once the feature selection algorithm was chosen and the best set of features were selected from PCA, we explored in the following section unsupervised learning process to compare with previous experiments carried out in [13].

### Clustering results after feature selection

**Fig. 5:**
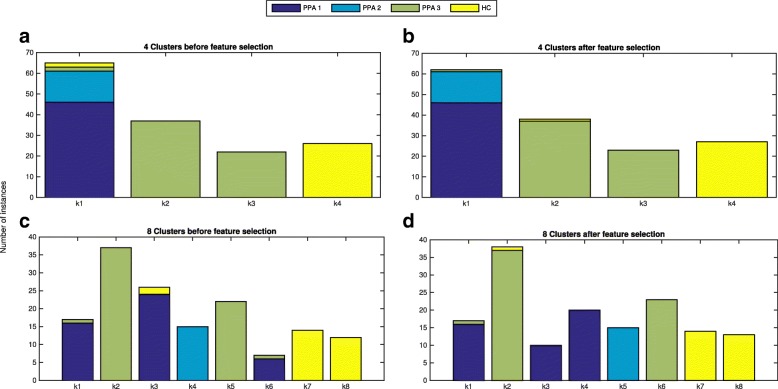
Clustering before/after feature selection except PCA. Distribution of patients within each cluster. X-axis represents the number of clusters, while in the Y-axis we show the number of patients assigned to each cluster. The different colors of the bars indicate the clinical PPA diagnosis for each patient within the cluster (1 nonfluent/agrammatic (dark blue), 2 semantic (light blue), 3 logopenic (green)) and healthy controls (yellow)

In a previous work [13], we applied Agglomerative Hierarchical Clustering (AHC) as unsupervised learning algorithm [24], particularly the *Ward Linkage* [25] algorithm, to evaluate how patients were split into subgroups taking into account clusters from 4 to 8. According to the experimental results, the distribution of patients in 8 clusters led 6 different subtypes of PPA, what reinforces the latest clinical studies [26–28]. These experiments were carried out considering all attributes for classification. Figure 5 shows the distribution of patients within each cluster for 4 and 8 cluster, before and after the feature selection process. The X-axis is the number of clusters and Y-axis the number of patients belonged to each cluster. Figure 5a and c represent results obtained in the previous work, without feature selection. Figure 5b and d correspond to results obtained after the feature selection process. Different colors in bars represent the three subtypes currently recommended in consensus PPA criteria. Dark blue is not-fluent/agrammatic, light blue represents semantic, green corresponds to logopenic and controls are represented by the yellow color. Particularly, instances assigned for each cluster are shown in Table 3.

**Table 3:** Distribution of patients per cluster and clinical PPA diagnosis for *Linkage* and 4 and 8 cluster

	

Parts labeled as *a* and *c* correspond to results before the feature selection process, whereas *b* and *d* represent results with the set features selected above. Number of patients assigned to each cluster are shown, and their previous clinical PPA diagnosis is represented by the column labeled PPA. Values 1, 2 and 3 in the first column correspond to nonfluent/agrammatic, semantic and logopenic PPA, respectively, whereas *HC* represents healthy controls. *Kn* specifies the number of clusters, where **n=0,1,..,N** and *N* is a value between 4 and 8

After the feature selection phase, the expert neurologist evaluated four feature selection algorithms and he selected the *ClassifierAttributeEval* algorithm as the most suitable, hence the subset of features provided by this algorithm are considered as the most relevant. Therefore, we launched a new set of experiments to compare these results with those from the previous study.

We analyzed the *Linkage* clustering algorithm, and compared results before and after feature selection. Clustering results after the feature selection process for the *Linkage* clustering algorithm are outlined in Fig. 5b and d, where X-axis represents the number of clusters evaluated and Y-axis corresponds to the number of patients assigned to each cluster. The clinical PPA diagnosis for each patient is represented by a different color within the cluster. We provide a more detailed information of the distribution of patients per cluster in Table 3 *b* and *d*.

We compared the classification results for 4 and 8 clusters after feature selection with those obtained in our previous study. Regarding 4 clusters, the group k1 contains in both cases all no-fluent and semantic PPA patients, either after the classification performed with all attributes, and after feature selection. However, a logopenic patient is moved to k3 group, and two patients previously belonging to the healthy control are moved to k4 and k2, respectively.

With respect to 8 clusters, the first group k1 remains unchanged and it contains the same patients than k1 group with all attributes. It comprises 16 patients with non-fluent and 1 patient with logopenic variants. K2 group includes same patients as the former k2 group and one healthy control. This healthy control is the only difference between both results.

The former k3 group included 24 patients with non-fluent and 2 controls. After feature selection, k3 is subdivided into two subgroups k3 (n=10) and k4 (n=20).

Patients diagnosed with semantic PPA are identified in both experiments in a single group, k4 in the former study and k5 after feature selection. The k5 group after feature selection includes all patients, which were in the k5 group in the previous work. Moreover, one patient is moved to k5 when it belonged to the former k6. Finally, healthy controls were also subdivided in two subgroups k7 and k8. K7 comprises 14 patients all of then women, and k8 13 men patients. The only difference is given in the k8 group with a new patient moved from k3 in the previous study, where patients were mostly diagnosed with logopenic variants. As we previously mentioned, the subdivision of healthy controls in two groups probably reflects gender differences in regional brain metabolism, because 100% of cases in k7 and k8 were women and men, respectively [29].

Our results expose a finer grain classification of patients according to the neuroanatomy of their disease, and give a step further in the field of PPA diagnosis. However, with respect to the previous work the clustering analysis after feature selection had not profound improvements and we addressed the Principal Component Analysis (PCA)[30].

### Clustering after the principal components analysis

**Fig. 6:**
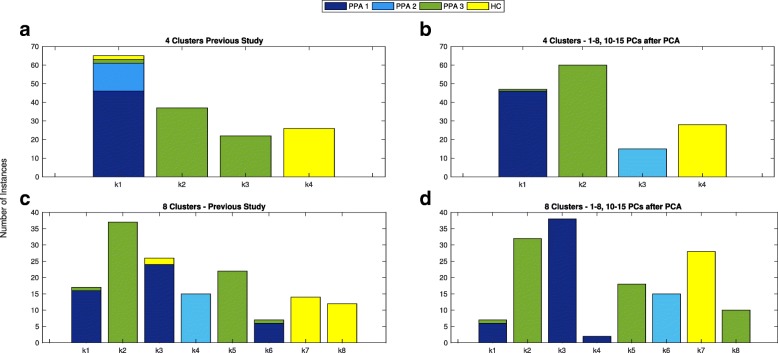
Clustering after PCA analyses. Distribution of patients within each cluster for Principal Components 1-8, 10-15. X-axis represents the number of clusters, while in the Y-axis we show the number of patients assigned to each cluster. The different colors of the bars indicate the clinical PPA diagnosis for each patient within the cluster (1 nonfluent/agrammatic (dark blue), 2 semantic (light blue), 3 logopenic (green)) and healthy controls (yellow). Clustering results for 4 and 8 clusters are drawn Fig. 6 in (**a**,**b**), and (**c**,**d**), respectively

After PCs with higher coverage of the variance were chosen, we launched the clustering analyses with those that reached better results in the classification process. Moreover, we tried to cover as many possibilities as possible, thus we tested different sets of PCs to indentify those features which better fit to the problem at hand. Results were compared with those from our previous study. Figure 6 displays the distribution of patients within each cluster, where X-axis represents the number of cluster and the Y-axis the number of patients assigned to each one. Colors corresponds to the clinical PPA diagnosis for patients within the cluster (1 nonfluent/agrammatic (dark blue), 2 semantic (light blue), 3 logopenic (green)) and healthy controls (yellow). More detailed information for 4 clusters is given at Table 4. Results from the study previously mentioned, labeled as (a), are detailed on the left (highlighted in bold). Next column, labeled as (b), represents the distribution of instances for the PCs selected. Clustering results for 4 and 8 clusters are drawn in Figures (a) and (b), and (c) and (d), respectively.

**Table 4:** Distribution of patients per cluster and clinical PPA diagnosis for *Linkage* and 4 and 8 clusters

	

Parts labeled as *a* and *c* correspond to results before the feature selection process, whereas *b* and *d* represent results with the set features selected above. Number of patients assigned to each cluster are shown, and their previous clinical PPA diagnosis is represented by the column labeled PPA. Values 1, 2 and 3 in the first column correspond to nonfluent/agrammatic, semantic and logopenic PPA, respectively, whereas *HC* represents healthy controls. *Kn* specifies the number of clusters, where **n=0,1,..,N** and *N* is 4

Regarding the column labeled as (b), which corresponds to experimental tests *1-8, 10-15* and *1-14 No Sex*, we observed that all except one instances are identified according to the standard PPA types. The group k1 contains all patients diagnosis as no-fluent and one instance with a prognosis as logopenic PPA. The remaining groups correspond to patients diagnosed with semantic and logopenic PPA, and controls, respectively. These results demonstrated that with a less number of features obtained from PCA, the most of instances are almost perfectly assigned according to the clinical diagnosis.

We focus on the clustering analyses for 8 clusters. Figure 6 shows the distribution of patients within clusters. We extended information in Table 4(b) and we compared results of the columns namely as (b) with the column labeled as (a).

Bellow is the analyses for **1-8,10-15 PCs (b)**. In this case the group k6 and k4 in (a) are identical to k1 and k6, respectively in (b). 6 instances from k5 and 12 from k2 in (a) are placed in the group k5 in (b). K1 and k3 in (a) distributed its patients between k3 and k4 in (b). Healthy controls were assigned to the only group k7 in (b).

## Discussion

Our study addresses the improvement of the PPA diagnosis from FDG-PET images applying machine learning techniques. Using results obtained in a previous work as a reference, we extend the study to identify the best classifier algorithm and the best set of features to simplify the PPA diagnosis. According to the classification results obtained with all features in phase 1, *SMO* followed by *IBK* are the best algorithms among those evaluated. Although twelve classifiers algorithms were selected, other algorithms can be evaluated in order to obtain the most precise results. Phase 2 was in charge to analyse the performance of four feature selection algorithms and PCA to identify the best set of features. This phase also considered the expert neurologist supervision and compared the results with those obtained in the phase 1. After this analysis, we cannot conclude that feature selection improves the number of instances correctly classified because the improvement percentage is not meaningful, although results are promising regarding the performance reached by some classifiers. Analysing a wider set of feature selection algorithms should allow not only to confirm these results but also reassert that other alternatives have to be explored.

According to the classification results obtained with the features extracted with PCA, we cannot generalize that PCA improves classification. There is no doubt that some classification algorithms equal or even improve the number of instances correctly classified with a less number of features.

Once the *ClassifierAttributeEval* algorithm selected the best set of features, we explored unsupervised learning with *Hierarchical Clustering* to compare with previous experiments carried out in [13]. Considering the set of features provided by the *ClassifierAttributeEval* algorithm, our results adjust the classification of patients according to the neuroanatomy of their disease, as we previously mentioned. Regarding the set of features from PCA, our results reinforce our previous outcomes with a less number of features. Most of the instances are almost perfectly assigned according to the clinical diagnosis. However, other machine learning branches can help to identify the best reduced set of relevant features and make easier the automation of the diagnosis of PPA.

## Conclusions

In this work, we have exploited machine learning techniques to improve the PPA diagnosis from FDG-PET images. This study confirms and reinforces the results obtained in a previous clinical work, where we explored the automatic classification of PPA patients and found out new subtypes of this disease that correlate with the clinical findings and better predict the clinical course. Here, we have proposed a machine learning approach that, on one hand, validate the clinical findings and, on the other hand, offers a practical and automatic tool to help on the diagnosis of PPA variants in order to improve the management of these patients. The data acquired from FDG-PET images are characterized by a high dimensionality, hence the application of the feature selection process is considered as a key proposal to reduce the dimensionality in order to improve the automatic classification. As part of the feature selection step, the expert knowledge has been included in a simplified and natural way for the clinical assessing, and could be automatized in the future by a set of rules. As a result, the number of patients correctly classified is increased after the most relevant features are identified, particularly after PCA analyses. As a conclusion, a fine grain classification has been obtained based on the neuroanatomy of the disease, which improves previous results for the PPA diagnosis. Overall, our study suggests the role of machine learning techniques applied to neuroimaging analysis in order to improve the classification of neurodegenerative diseases, and closes the gap between image processing and automatic diagnosis tools that help on the clinical practice.

## Methods

As aforementioned, our goal is to advance on the development of an automatic diagnosis tool based on machine learning approaches. For that purpose, we aim to improve the classification process carried out in [13] that brought the finding of new PPA variants. In the previously mentioned work, we used *Hierarchical clustering* as unsupervised learning algorithm [24], particularly the *Ward Linkage* [25] algorithm with all attributes present in the dataset. We concluded that unsupervised clustering analysis of FDG-PET data favored, based on the Davies-Bouldin index, the classification of PPA into six variants rather than three subtypes as currently recommended in consensus PPA criteria. However, input data are multidimensional; we have 150 instances and 119 attributes for each one and some attributes may introduce noise, which affects the classification process.

In this light, we considered necessary to identify the most relevant attributes, reducing the multidimensionality and improving the classification process, accurately. Figure 1 shows the flow chart proposed as methodology, which will be detailed below.

The first phase, identified as 1 in the flow chart, provides the dataset with all features to the classification phase, identified as 4, where for each classifier algorithm the process is launched. The set of selected classifiers was *BayesNet*, *NaivesBayes*, *SMO*, *IBK*, *Kstar*, *LWL*, *DecisionTable*, *OneR*, *PART*, *J48*, *DecisionStump* and *RepTree* from the machine learning software WEKA (Waikato Environment for Knowledge Analysis) [31] in order to evaluate which classifiers perform better for the problem of improving the diagnosis of PPA from FDG-PET images. As a result, we obtained the classification with all features. Phase labeled as 2 is in charge of the feature selection phase and consists in pinpointing those attributes considered more relevant to the problem at hand (this is to improve the classification of patients according to more accurate subtypes of PPA). For this phase, we selected four typical feature selection algorithms:*CfsSubsetEval*, *ChiSquardAttributeEval*, *ClassifierAttributeEval* and *WrapperSubsetEval*, and we applied the Principal Components Analysis (PCA) algorithm [30], although they are not comparable. PCA is a powerful mathematical algorithm for analysing data and reducing high dimensionality, preserving its variability as much as possible, and at the same time improving the data interpretability. PCA explores a dataset represented by a matrix *X*, where each row is one entity *n* and each column identifies a numerical variable *p*. The algorithm search the linear combination of the columns, which maximizes the variance. This algorithm is able to identify patterns in data and discover their differences and similarities to find the more relevant features. We applied PCA on the dataset previously mentioned. When the process is completed, a set of principal components is obtained. Next step, we decided to ignore the principal components of lesser significance in order to preserve as much information as possible.

Datasets obtained from the selected algorithms contain the most relevant features according to each algorithm. These datasets are used as input in the following phases, classification and clustering phases, labeled as 4 and 6, respectively.

The next phase corresponds to the clustering phase, identified as 6, where clustering analysis [32] is performed. In this phase, *Hierarchical clustering* [24] parameterized to Ward’s Linkage is launched. Instances are classified in different groups. The result of this process is compared against the clusters found in our previous work, 8 clusters, and also against the clusters described in the classical medical literature, 4 clusters.

## Data Availability

Al data are available in a systematic Data Base created by the Department of Neurology of the San Carlos Hospital, in Madrid, and accessible to clinicians and researchers participating in the project. These data are not publicly available due to data privacy laws.

## Abbreviations

AHC: Agglomerative hierarchical clustering
FDG-PET: Fluorodeoxyglucose positron emission tomography
FPR: False positive rate
IBK: Instance based learner K-nearest
KStar: Search algorithm K*
lvPPA: Logopenic variant PPA
LWL: Locally weighted learning
MRI: Magnetic resonance imaging
MS: Mass spectrometry
PART: Partial decision trees
PC: Principal components
PCA: Principal components analisys
PET: Positron emission tomography
PPA: Primary progressive aphasia
ROC: Receiver operating characteristics
SMO: Sequential minimal optimization
TDP-43: TAR-DNA binding protein-43
TPR: True positive rate
